# Supplementary material for: Hydralazine induces stress resistance and extends C. elegans lifespan by activating the NRF2/SKN-1 signalling pathway
Source: Nat Commun. 2017 Dec 20;8:2223. doi: 10.1038/s41467-017-02394-3 (PMC5738364; doi:10.1038/s41467-017-02394-3)
Supplement: Supplementary file 1 — Supplementary Information [file 41467_2017_2394_MOESM1_ESM.pdf]

a Cytoplasm

b Nucleus

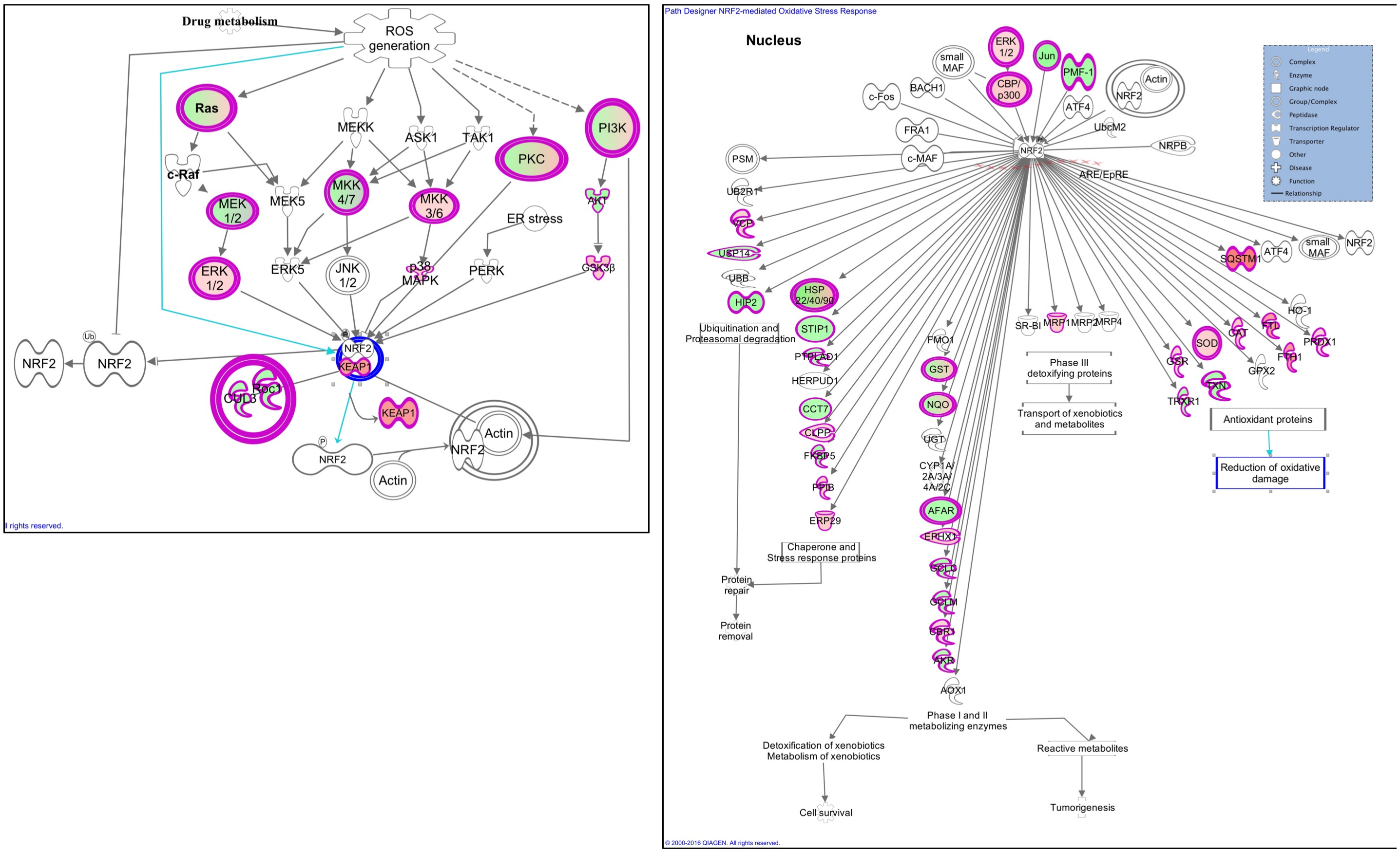

Supplementary Figure 1. NRF2 pathway activation identified with IPA using protein ratios determined using shotgun analysis of SH-SY5Y cells grown in SILAC media and treated with hydralazine. (a) NRF2 pathway activation in the cytoplasm. **(b)** NRF2 pathway activation in the nucleus. For shape and color codes follow this link. (http://ingenuity.force.com/ipa/articles/Feature_Description/Legend).

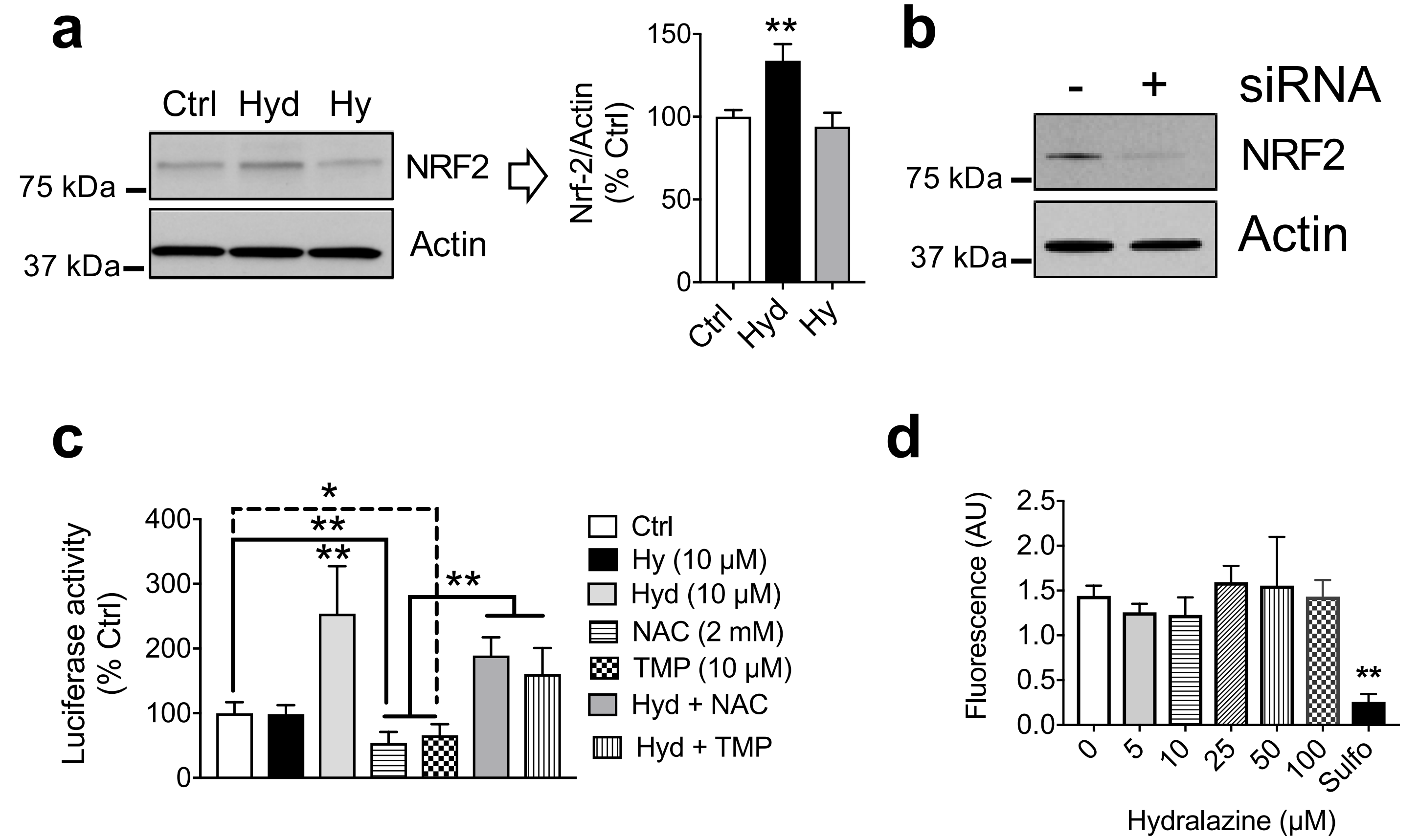

Supplementary Figure 2. Validating NRF2 activation and exploring its mechanism of action

(a) Hydrazine used as negative control for hydralazine did not increase NRF2 protein measured by Western blot analysis. Both hydralazine and hydrazine were used at 5 μM concentration. **(b)** Confirming the specificity of the antibody used for NRF2 Western blot analysis using NRF2 knockdown SH-SY5Y cells. **(c)** The ARE-driven luciferase activity was decreased in SH-SY5Y cells treated with antioxidant compounds N-acetyl cysteine (NAC, 2 mM) and Tempol (TMP, 10 μM) and increased when cells were treated with the antioxidant compounds and hydralazine (10 μM) indicating that hydralazine-mediated NRF2 activation was ROS independent, $^{*}p<0.05$ and $^{**}p<0.01$, student t-test, n=3, mean ± SD. **(d)** KEAP1-NRF2 Inhibitor Screening Assay showed that hydralazine dose not directly interrupt the interaction between NRF2 and KEAP1. $^{**}p<0.01$ student t-test, n=3, mean ± SD. Sulforaphane (5 μM) was used as a positive control (inhibitor), $^{**}p<0.01$, student t-test, n=3, mean ± SD.

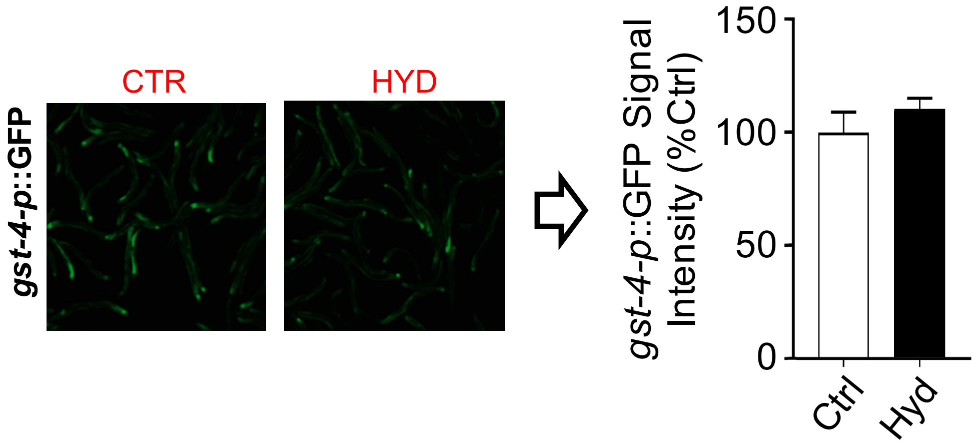

Supplementary Figure 3. Testing a downstream target of SKN-1 in a mutant strain lacking functional SKN-1C. Hydralazine treatment did not induce *gst-4p*::GFP expression in transgenic worms (*dvIs19*) lacking a functional SKN-1 isoform C in their intestine in a mutant background *skn-1(zu67).* $p>0.05$ student t-test, $n=50$ two independent trials, mean ± SD.

a **b**

Cytoplasm Nucleus

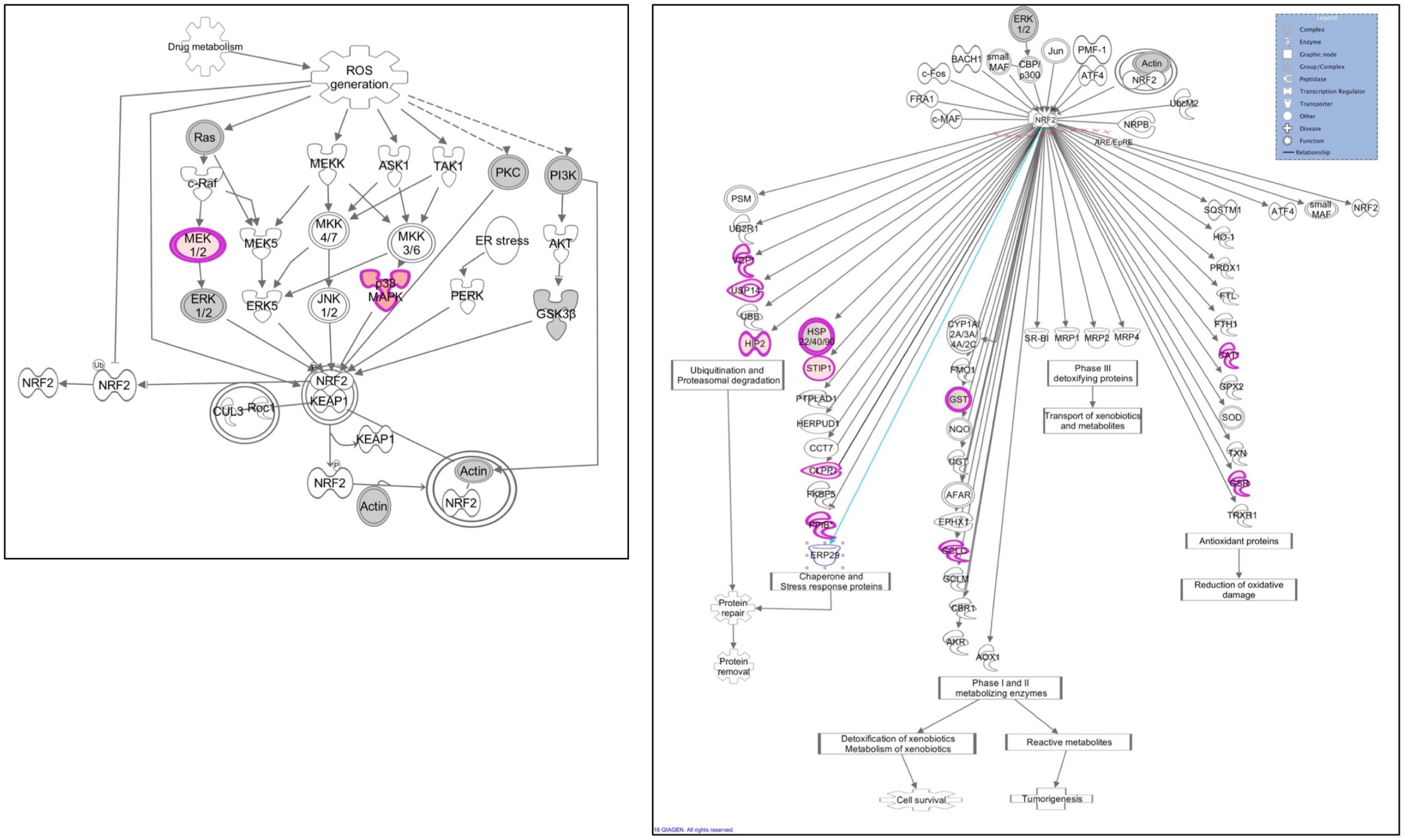

Supplementary Figure 4. SKN-1/NRF2 pathway activation identified with IPA using protein ratios determined using label free analysis of wild type *C. elegans* treated with hydralazine or vehicle. (a) SKN-1/NRF2 pathway activation in the cytoplasm. **(b)** SKN-1/NRF2 pathway activation in the nucleus. For shape and color codes follow this link (http://ingenuity.force.com/ipa/articles/Feature_Description/Legend).

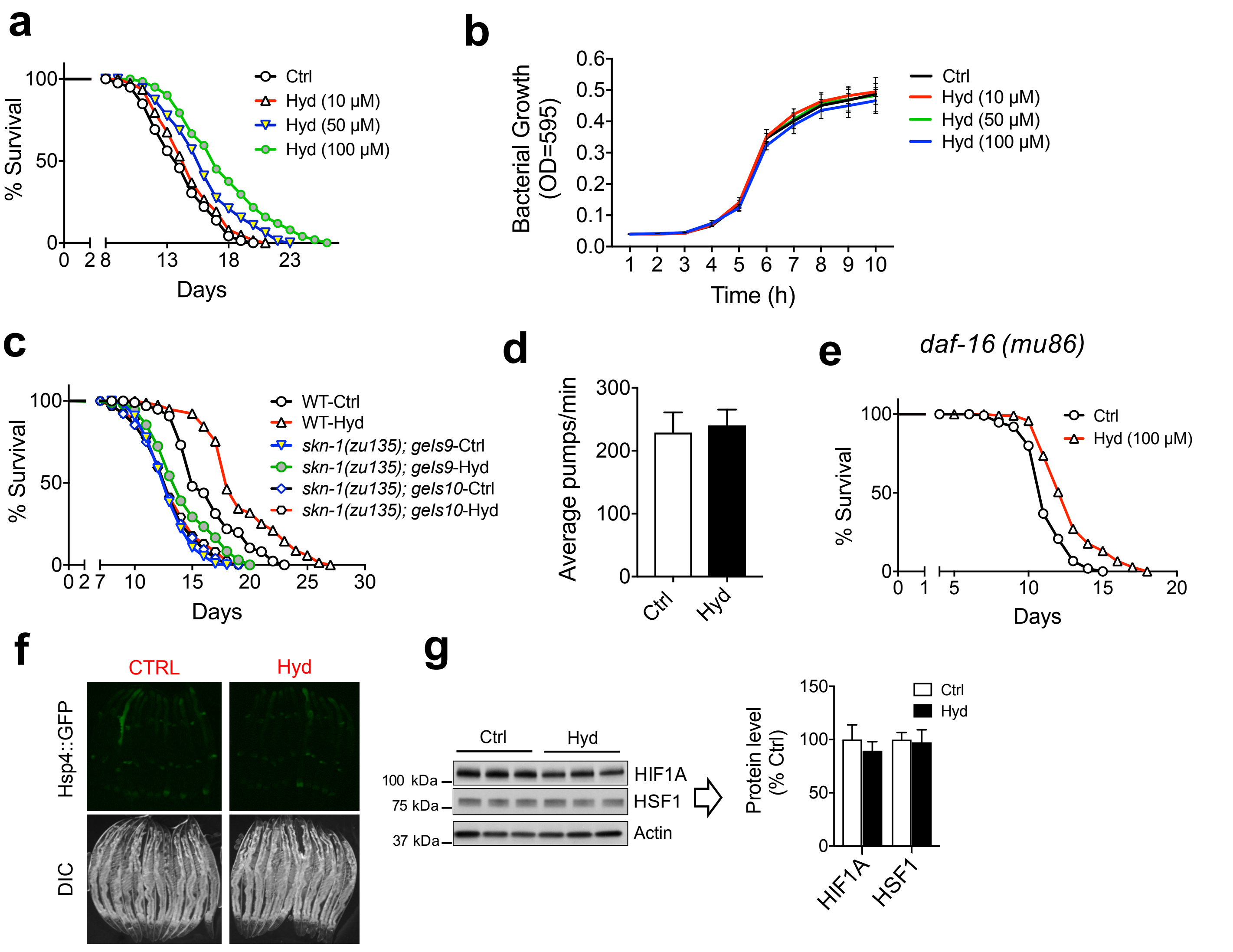

Supplementary Figure 5. Hydralazine extends lifespan in *C. elegans.* For all lifespan statistics, see Supplementary Table 3. (a) Hydralazine treatment increased *C. elegans* lifespan in a dose-dependent manner. **(b)** HB101 bacteria in liquid LB treated with different concentrations of hydralazine did not show growth retardation, two-tailed student t-test, n=6. **(c)** Expression of *skn-1* isoforms *b* by transgene *geIs9* partially restores longevity benefits of hydralazine (100 μM) while expression of isoform *c* in transgenic *geIs10* did not, signifying the role of *skn-1* isoforms *b* in hydralazine-mediated lifespan extension. **(d)** Pharyngeal pumping rate of wild type young (day 4) *C. elegans* treated with 100 μM hydralazine was not significantly altered, rulings out the possibility of hydralazine interfering with food uptake mimicking calorie restriction, two-tailed student t-test, n=32, mean ± SD. **(e)** Hydralazine mediated lifespan extension was not significantly attenuated in mutant *daf-16 C. elegans* ruling out the possibility of daf-2 insulin/IGF-1 signaling pathway involvement in prolongevity effects of hydralazine. **(f)** Fluorescence microscopic images tracing *hsp-4p::GFP* protein, a reporter of UPR^{ER} activation, indicated that hydralazine does not induce ER stress. **(g)** Two other important regulators of aging paradigm, HIF1A and HSF1, did not change in SH-SY5Y cells treated with 10 μM hydralazine. Two tailed student t-test, n=3, mean ± SD.

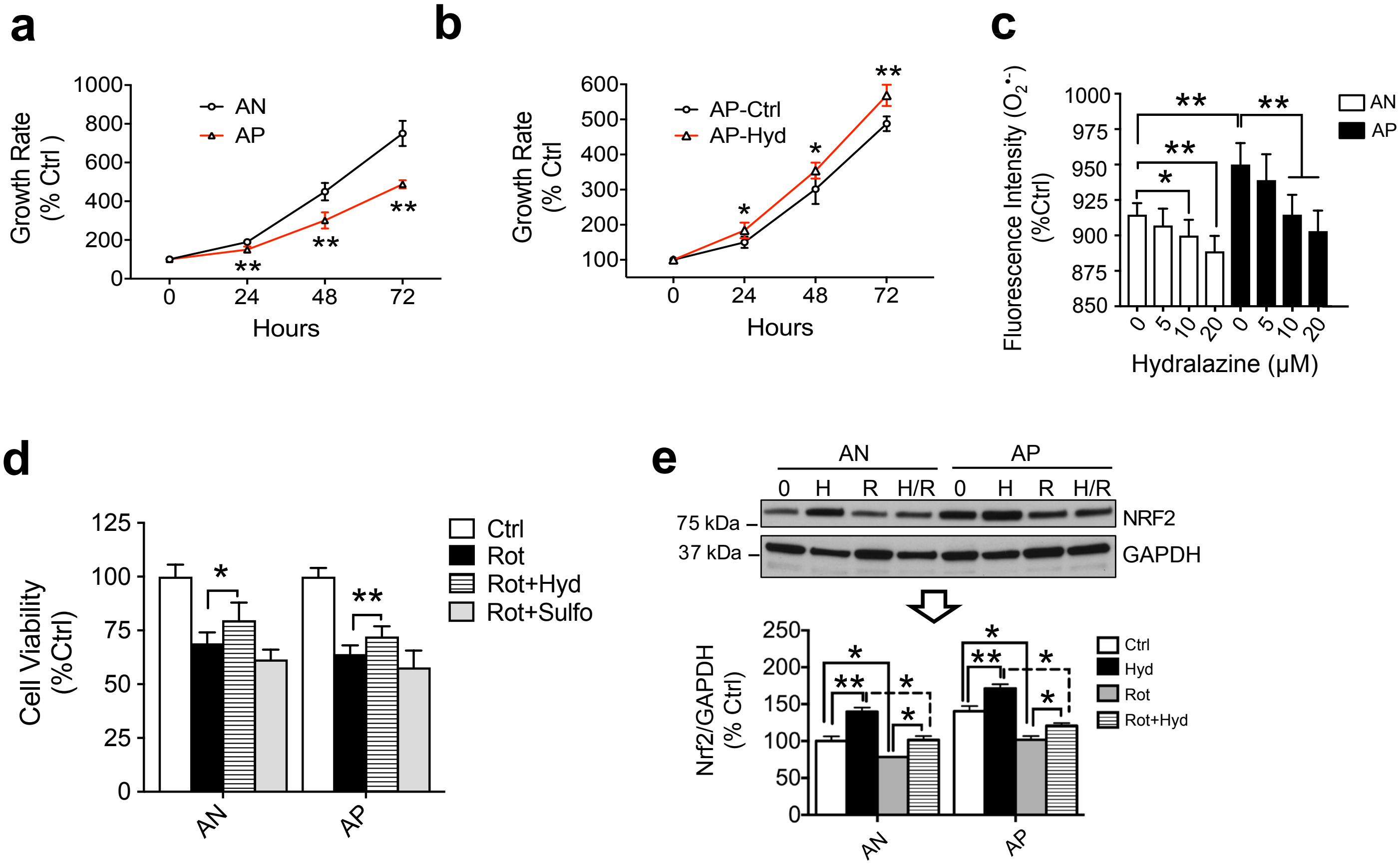

Supplementary Figure 6. Hydralazine protects tauopathy model cells from exogenous and endogenous stressors presenting a therapeutic potential for the treatments of neurodegenerative diseases. (a) Cell growth analysis showed a slower growth rate for aggregate-positive (AP) cells compared to control aggregate-negative (AN) cells. **p<0.01 student t-test, n=8, mean ± SD. **(b)** Hydralazine treatment (5 µM) improves the growth rate of aggregate-positive cells. *p<0.05 and **p<0.01, student t-test, n=8, mean ± SD. **(c)** Superoxide fluorescence signal intensity was higher in aggregate-positive cells compared to the control cells and decreases with hydralazine treatment in a dose dependent-manner in both cell models. *p<0.05 and **p<0.01, student t-test, n=6, mean ± SD. **(d)** Hydralazine (10 µM) improved the viability of both aggregate-positive and negative cells that are under rotenone stress (1 µM). Sulforaphane (5 µM) was used as positive control. **p<0.01, student t-test, n=6, mean ± SD. **(e)** Hydralazine (10 µM) reversed rotenone-mediated (1 µM) reduction in NRF2 protein signal intensity measured by Western blot. *p<0.05 and **p<0.01, student t-test, n=3, mean ± SD.

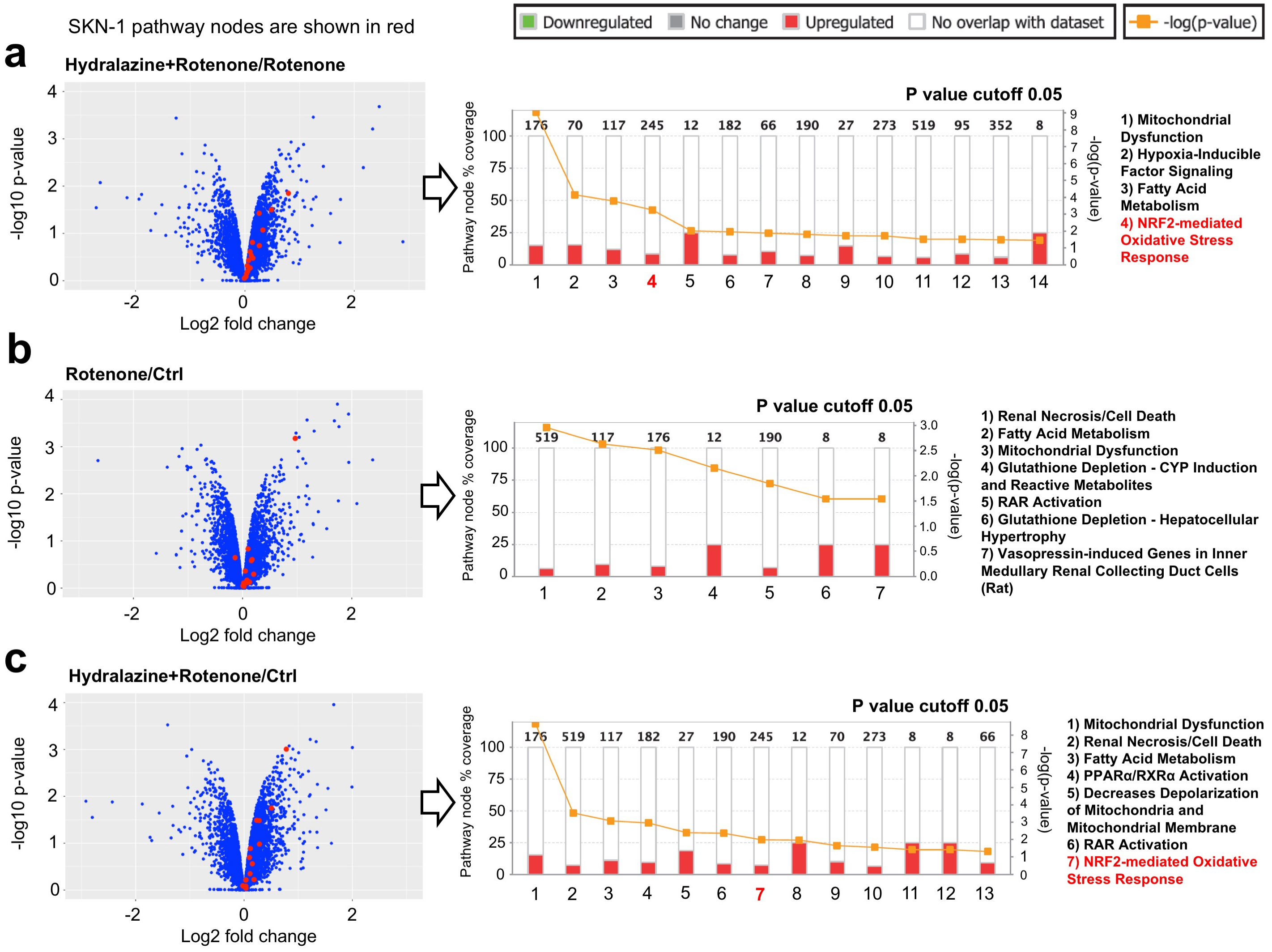

Supplementary Figure 7. Hydralazine activates SKN-1/NRF2 pathway in worms treated with rotenone. **(a)** Volcano plot showing the ratio (hydralazine+rotenone/rotenone) distribution of proteins quantified by label-free mass spectrometry. Proteins ratios obtained by label free mass spectrometry were uploaded for IPA analysis. SKN-1/NRF2 was number four in the top five activated stress response pathways (p-value cutoff of 0.05, right-tailed Fisher Exact Test.). **(b)** Volcano plot showing the ratio (rotenone/Ctrl) distribution of proteins quantified by label-free mass spectrometry. The results of IPA analysis for worms treated with rotenone compared to control. SKN-1/NRF2 was not among activated pathways (p-value cutoff of 0.05, right-tailed Fisher Exact Test.). **(c)** Volcano plot showing the hydralazine+rotenone/Ctrl ratio distribution of proteins quantified by label-free mass spectrometry. SKN-1/NRF2 pathway was among activated pathways when worms treated with rotenone and hydralazine were compared to control worms (p-value cutoff of 0.05, right-tailed Fisher Exact Test.). We showed our results pathways based on the p-value simply because this is a statistical figure of-merit. From a statistical point of view, a p-value means that the probability of getting the results obtained with a set null hypothesis (in our case was whether the results are statistically significantly different or not) is true. This is an indication for reproducibility. Further, these results were confirmed with the volcano plot based on Tukey Honestly Significant difference Test.

Supplementary Figure 8:

Figure 3a.

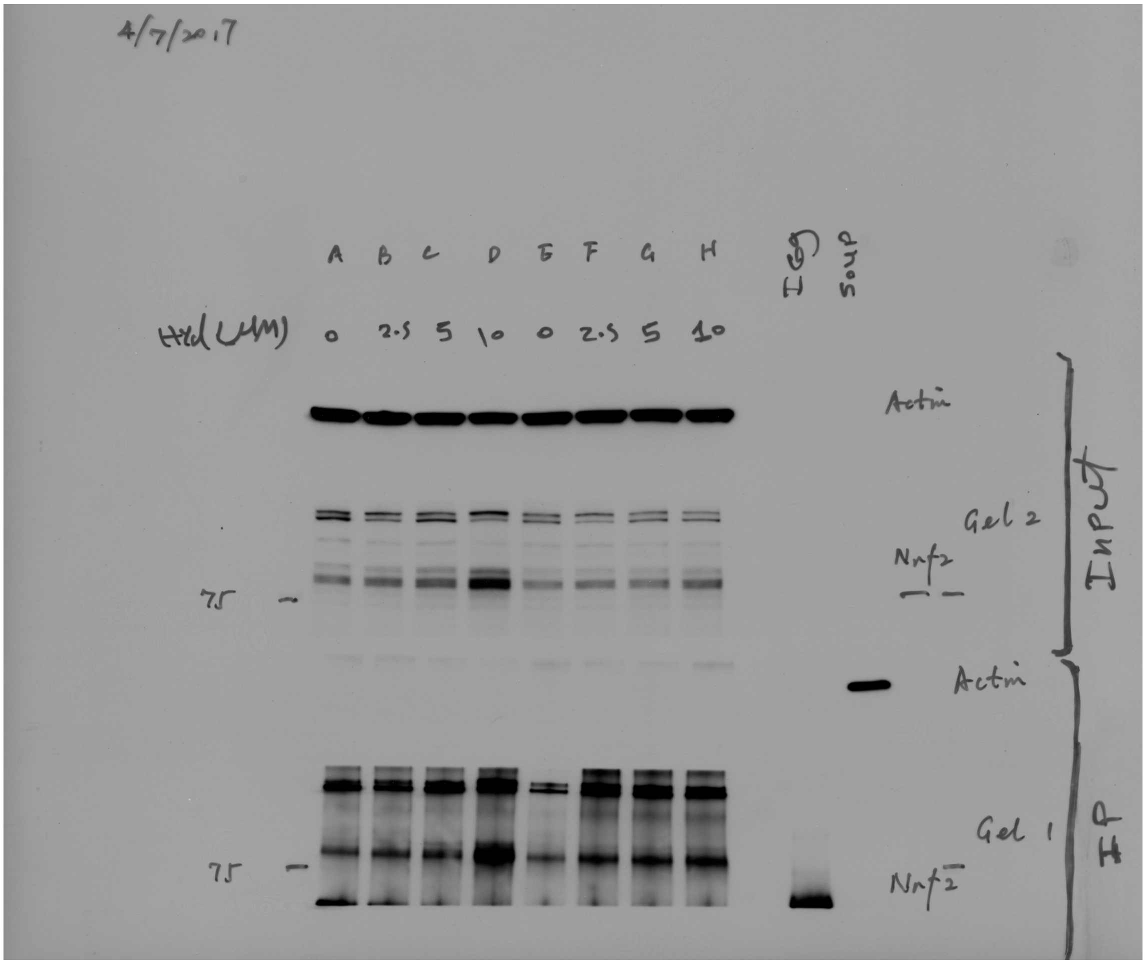

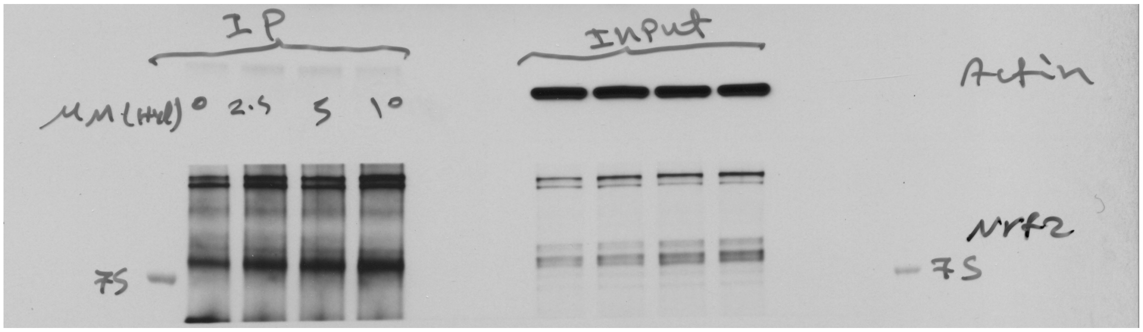

Figure 3c.

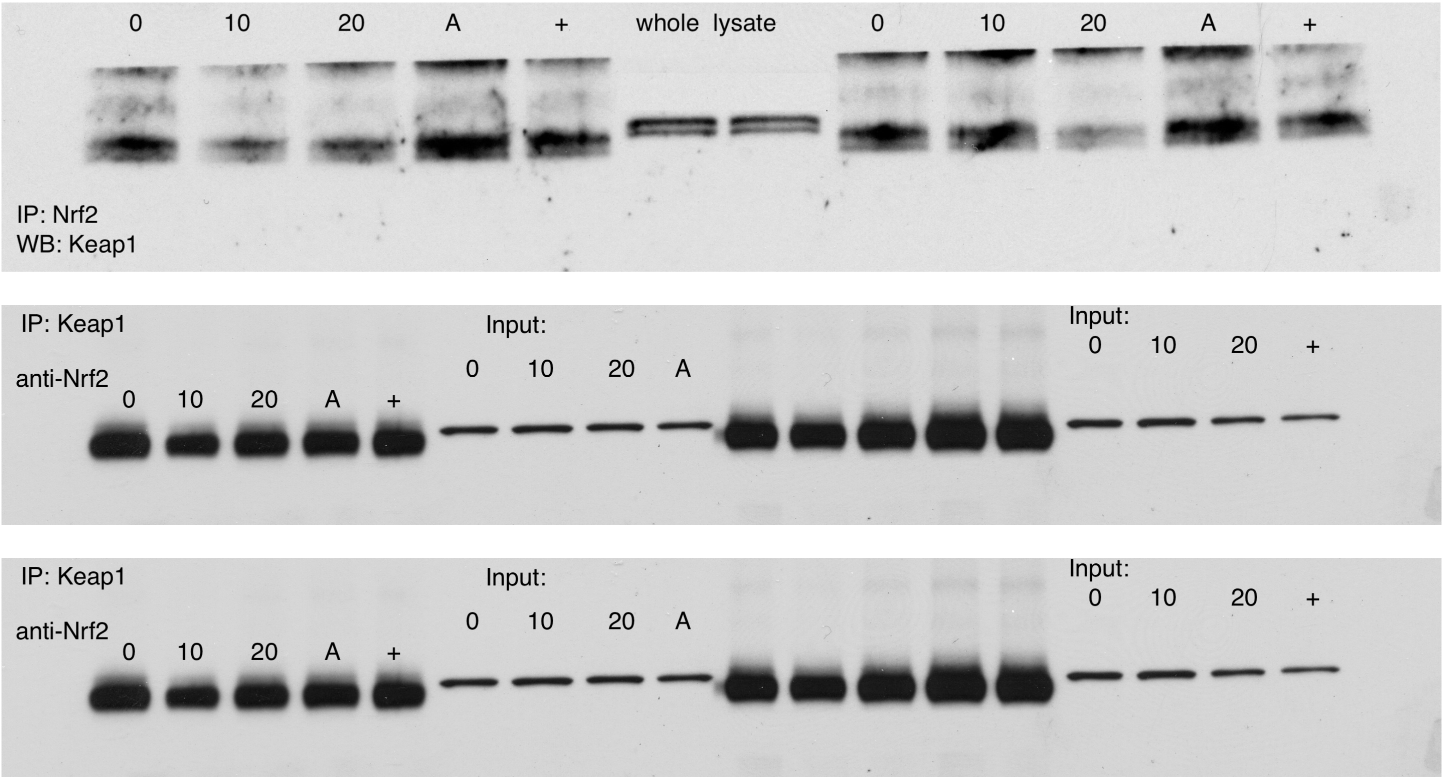
0 10 20 A + whole lysate 0 10 20 A +
IP: Nrf2
WB: Keap1
IP: Keap1
anti-Nrf2
0 10 20 A +
Input:
0 10 20 A
Input:
0 10 20 +
IP: Keap1
anti-Nrf2
0 10 20 A +
Input:
0 10 20 A
Input:
0 10 20 +

Figure 3d.

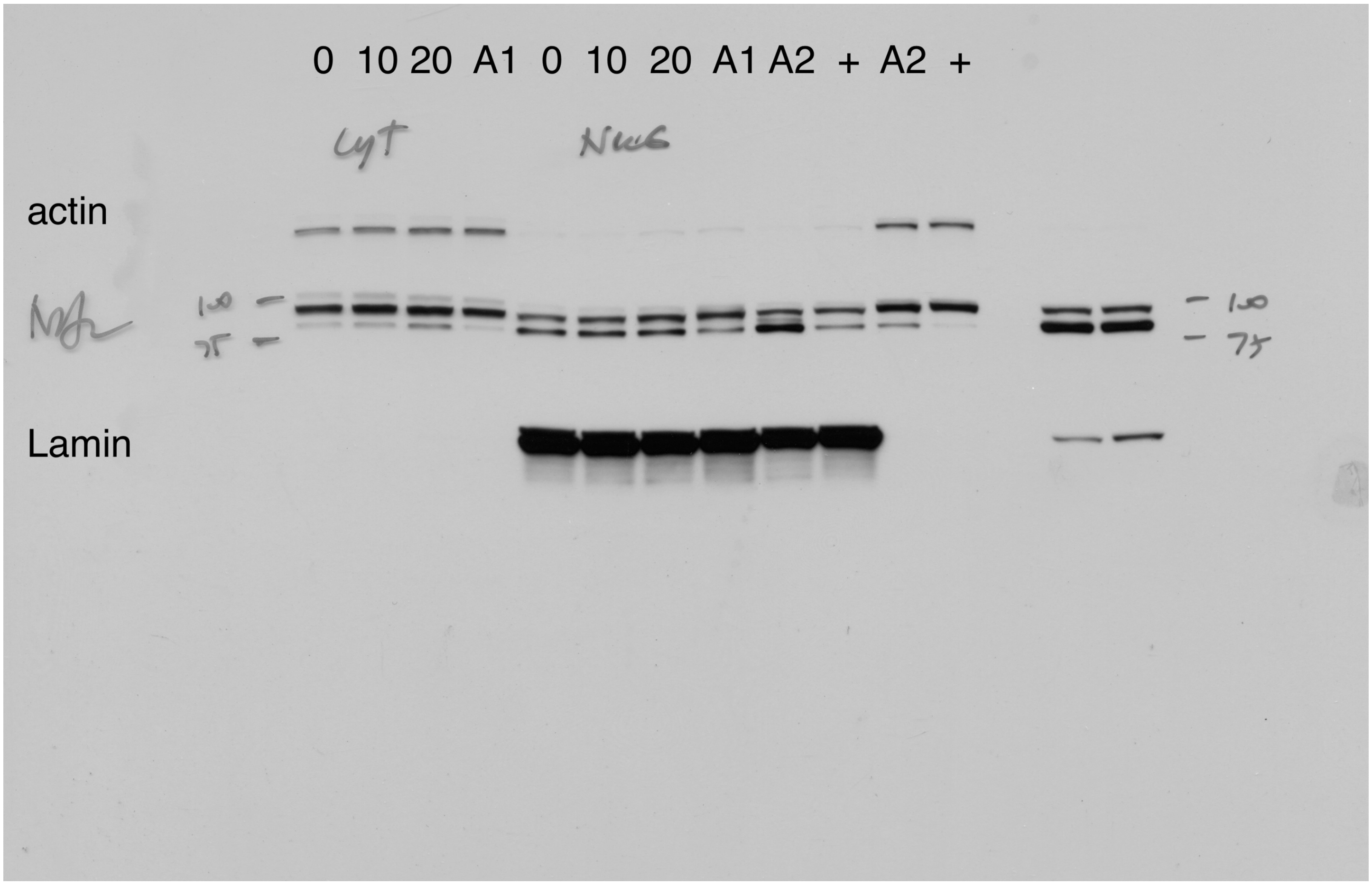

Figure 3e.

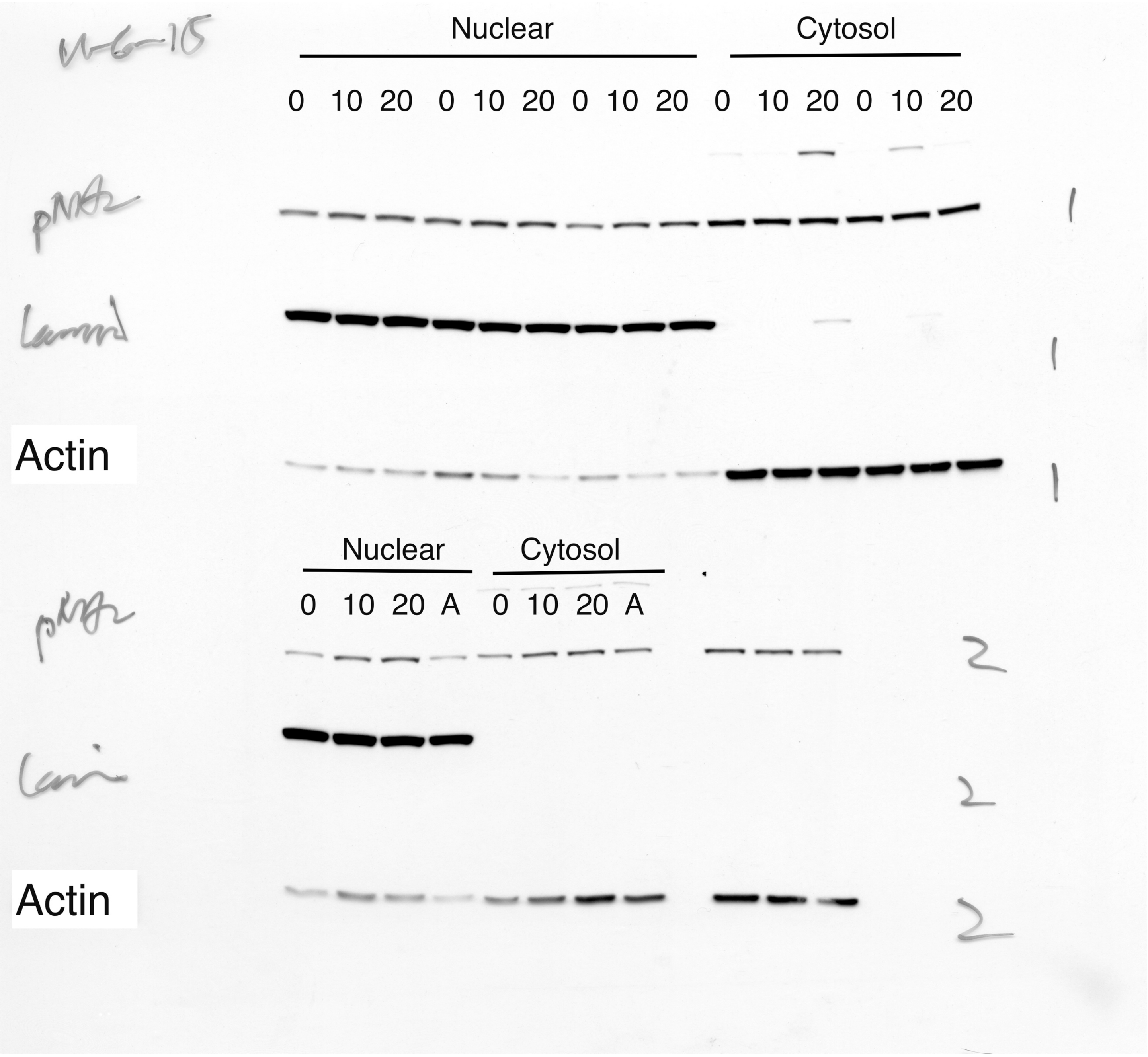
Nuclear
Cytosol
0 10 20 0 10 20 0 10 20 0 10 20 0 10 20
Actin
Nuclear
Cytosol
0 10 20 A 0 10 20 A
Actin

Figure 3f.

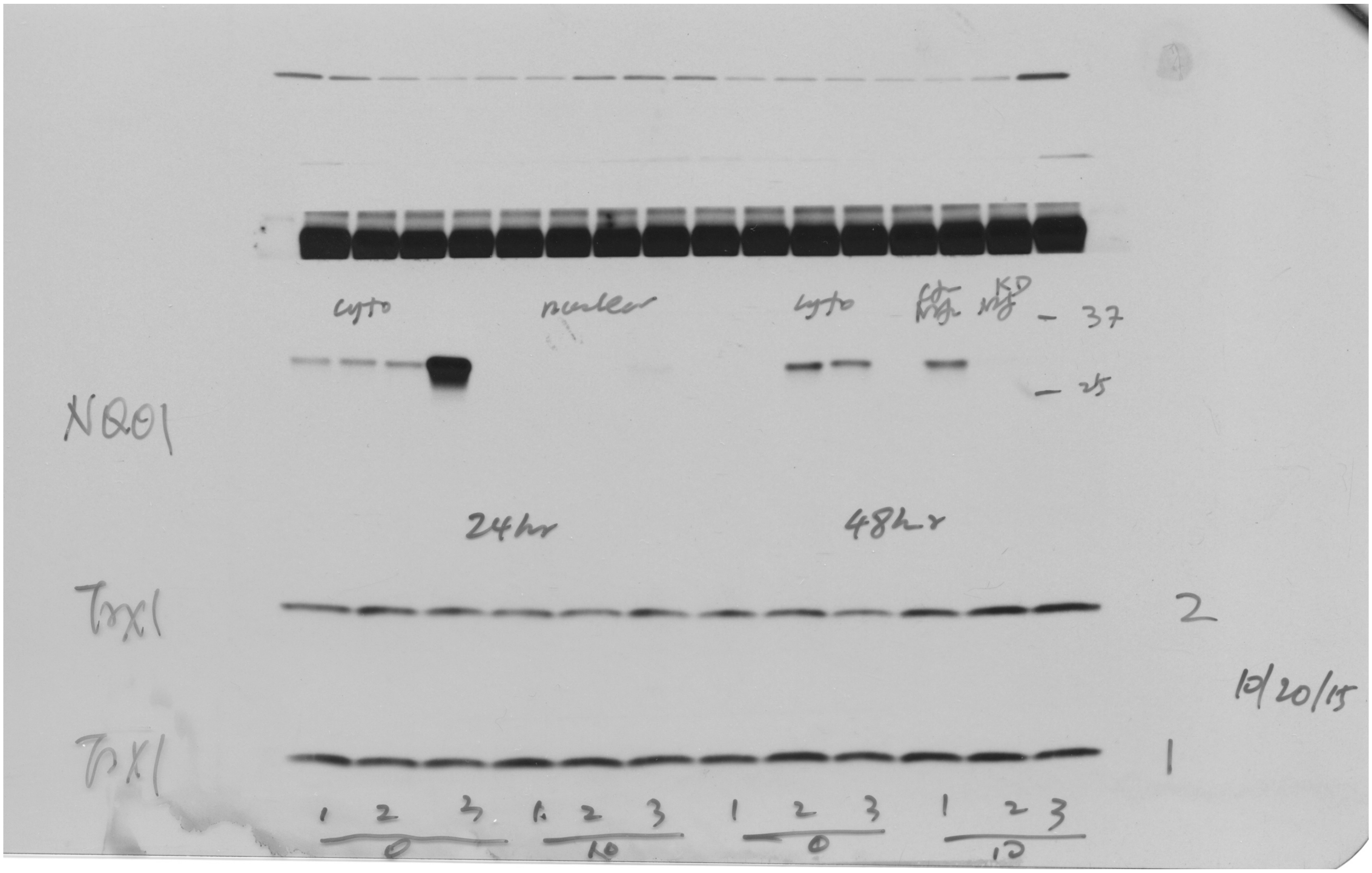
cyto
nuclear
cyto
- 37
- 25
NQO1
24hr
48hr
TrX1
2
10/20/15
TrX1
1
1 2 3
0
1 2 3
10
1 2 3
0
1 2 3
10

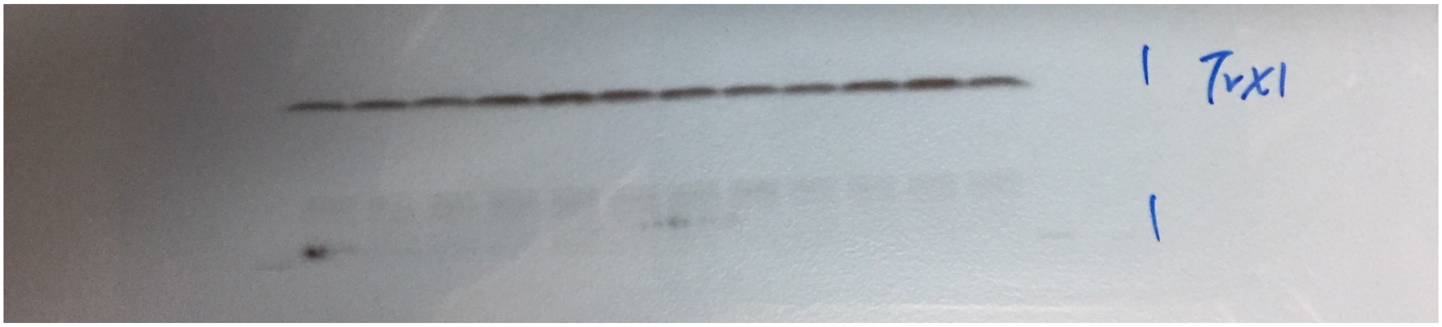
1 TrX1
1

Figure 3i.

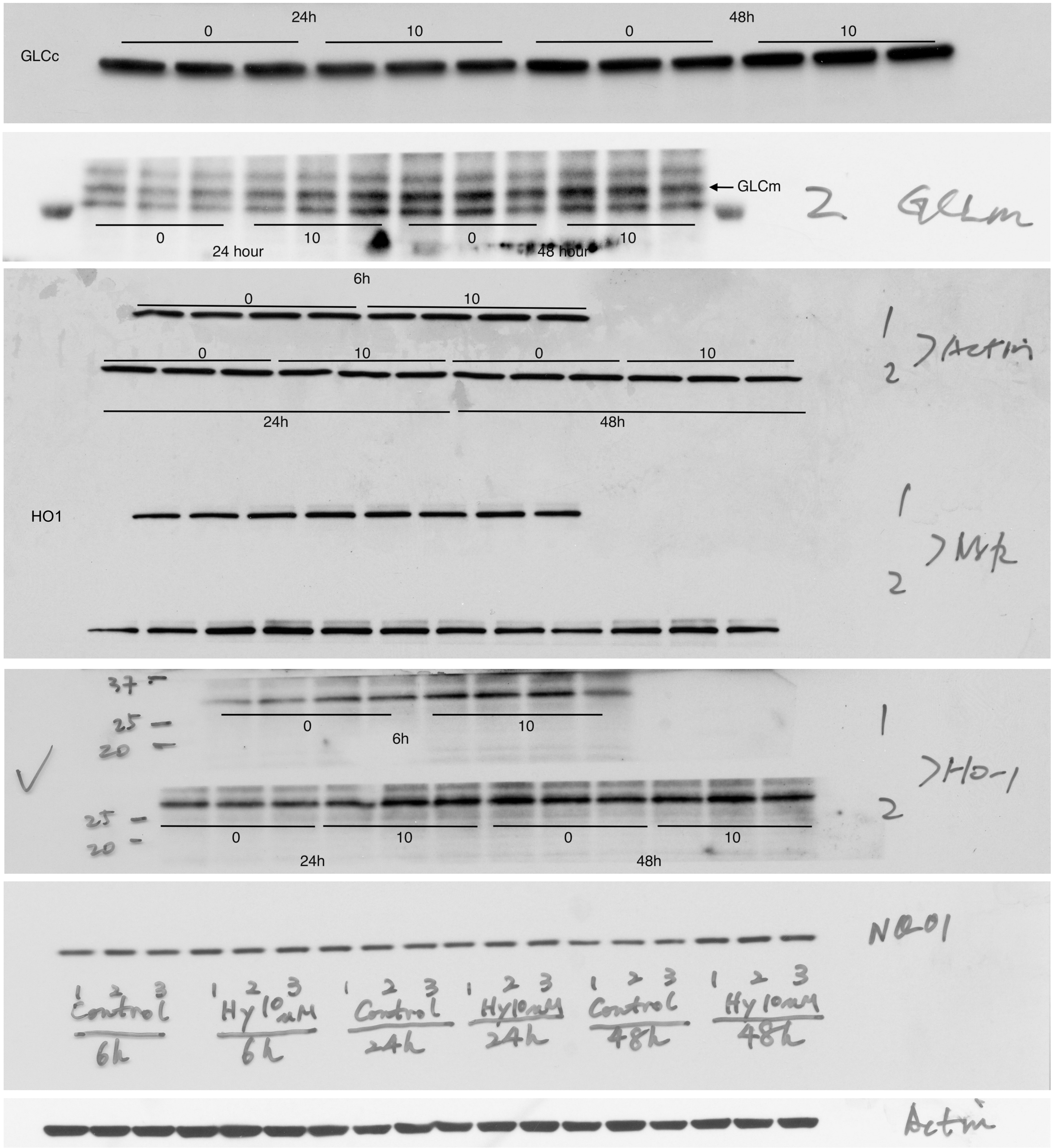
GLCc
24h
48h
0
10
GLCm
2 GLCm
24 hour
48 hour
6h
1
2
Actin
24h
48h
HO1
Nrf2
37
25
20
HO-1
NQO1
1 2 3 Control 6h
1 2 3 Hy 10 mM 6h
1 2 3 Control 24h
1 2 3 Hy 10mM 24h
1 2 3 Control 48h
1 2 3 Hy 10mM 48h
Actin

Supplementary Table 1. Sequences of primers used for quantitative real-time PCR analysis of NRF2 pathway gene expression.

Gene name	Forward (5'-3')	Reverse (5'-3')	Annealing temperature (°C)
NRF2	**AACCACCCTGAAAGCACAGC**	**TGAAATGCCGGAGTCAGAATC**	60
NQO1	**CGCAGACCTTGTGATATTCCAG**	**CGTTTCTTCCATCCTTCCAGG**	60
HMOX1	**TCTCTTGGCTGGCTTCCTTAC**	**GCTTTTGGAGGTTTGAGACA**	60
GST4	**GAGAACCCTGATTGACATGTA**	**GCTGATTACCAACAAGAAAGC**	60
GSTP1	**TCCCTCATCTACACCAACTATGAG**	**GGTCTTGCCTCCCTGGTT**	60
GCLC	**ATGGAGGTGCAATTAACAGAC**	**ACTGCATTGCCACCTTTGCA**	60
GCLM	**GCTGTATCAGTGGGCACAG**	**CGCTTGAATGTCAGGAATGC**	60
β-actin	**GCCGGGACCTGACTGACTAC**	**TTCTCCTTAATGTCACGCACGAT**	60

Supplementary Table 2. Statistical data for lifespan studies.

Correspon ding Figure	Strain, treatment	Median Lifespan (days)		% difference	P-values	N (number of animals)	
		Vehicle	Treatment			Vehicle	Treatment
Fig. 5a and Suppl. Fig 5a	N2, 10 µM Hyd	14	15	+07.14	0.3494	105	108
	N2, 50 µM Hyd	13<br>14	15<br>16	+15.38<br>+14.28	<0.0001<br><0.0001	86<br>105	98<br>112
	N2, 100 µM Hyd	14<br>13<br>15<br>14	17<br>16<br>18<br>18	+21.50<br>+23.07<br>+20.00<br>+28.60	<0.0001<br><0.0001<br><0.0001<br><0.0001	105<br>86<br>75<br>108	109<br>88<br>84<br>112
Fig. 5b	N2-heat-inactivated 100 µM Hyd	18<br>17	22<br>21	+22.22<br>+23.52	<0.0001<br><0.0001	117<br>102	120<br>95
	N2- 100 µM Hyd pretreated HB101	15	15	-	02696	99	90
Fig. 5c	N2,100 µM Hyd	15<br>14	18<br>16	+20.00<br>+14.28	<0.0001<br><0.0001	130<br>117	112<br>101
	N2,100 µM Curcumin	15	16	+06.25	0.0815	130	122
	N2,20 mM Metformin	15<br>14	17<br>16	+13.30<br>+14.28	<0.0006<br><0.0001	130<br>117	99<br>120
Fig. 5d	N2- Scr. *RNAi*,100 µM Hyd	13<br>14	16<br>17	+23.07<br>+21.50	<0.0001<br><0.0001	79<br>87	80<br>84
	N2- *skn-1 RNAi*, 100 µM Hyd	10<br>12	09<br>12	-10.00<br>-	0.0199<br>0.9684	89<br>94	84<br>102
Fig. 5e	EU31, 100 µM Hyd	10<br>11	10<br>11	-<br>-	0.3257<br>0.4184	80<br>112	95<br>108
	EU1,100 µM Hyd	11<br>13	12<br>15	+09.09<br>+16.16	0.0141<br><0.0001	91<br>103	105<br>97
Fig. 5g	DA1113,100 µM Hyd	22<br>24	21<br>24	-04.54<br>-	0.4118<br>0.2646	70<br>82	67<br>83
Suppl. Fig. 5c	LG357,100 µM Hyd	13<br>11<br>12	13<br>11<br>13	-<br>-<br>+08.33	0.3820<br>0.3628<br>0.1946	90<br>108<br>94	95<br>115<br>93
	LG348,100 µM Hyd	14<br>12<br>11	16<br>13<br>12	+14.28<br>+08.33<br>+09.09	<0.0001<br>0.0868<br>0.0304	108<br>68<br>77	101<br>69<br>75
Suppl. Fig. 5e	CF1038,100 µM Hyd	10<br>11	12<br>13	+20.00<br>+18.18	<0.0001<br><0.0001	56<br>64	60<br>64
Fig. 6d	BR6516, 100 µM Hyd	13	17	+30.76	<0.0006	64	60
	BR5270,100 µM Hyd	08<br>10<br>11	11<br>13<br>13	+37.50<br>+30.00<br>+18.00	<0.0001<br><0.0001<br><0.0001	106<br>71<br>114	105<br>75<br>112
