# Supplementary material for: Hydralazine induces stress resistance and extends C. elegans lifespan by activating the NRF2/SKN-1 signalling pathway
Source: Nat Commun. 2017 Dec 20;8:2223. doi: 10.1038/s41467-017-02394-3 (PMC5738364; doi:10.1038/s41467-017-02394-3)
Supplement: Supplementary file 3 — Description of Additional Supplementary Files [file 41467_2017_2394_MOESM3_ESM.docx]

**Description of Additional Supplementary Files**

File Name: Supplementary Data 1

Description: List of pathways activated/deactivated in SH-SY5Y celles treated with 10 µM hydrazine generated by Ingenuity Pathway Analysis
